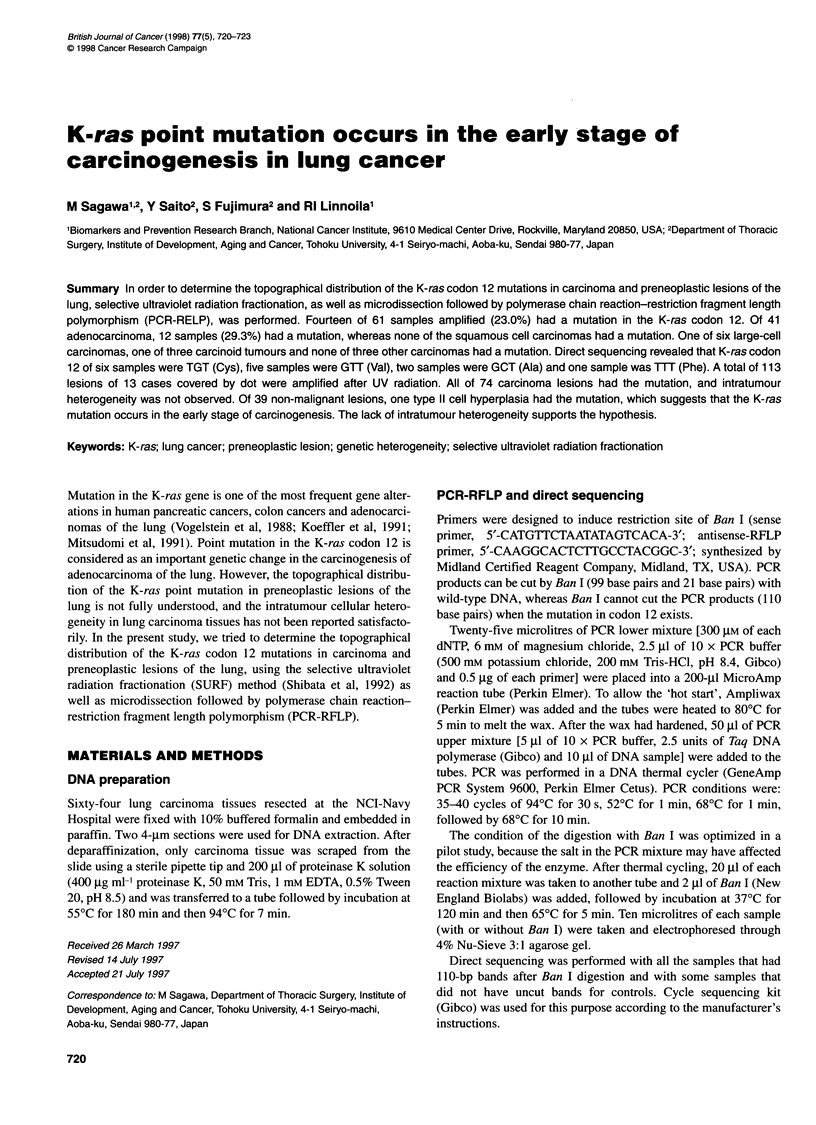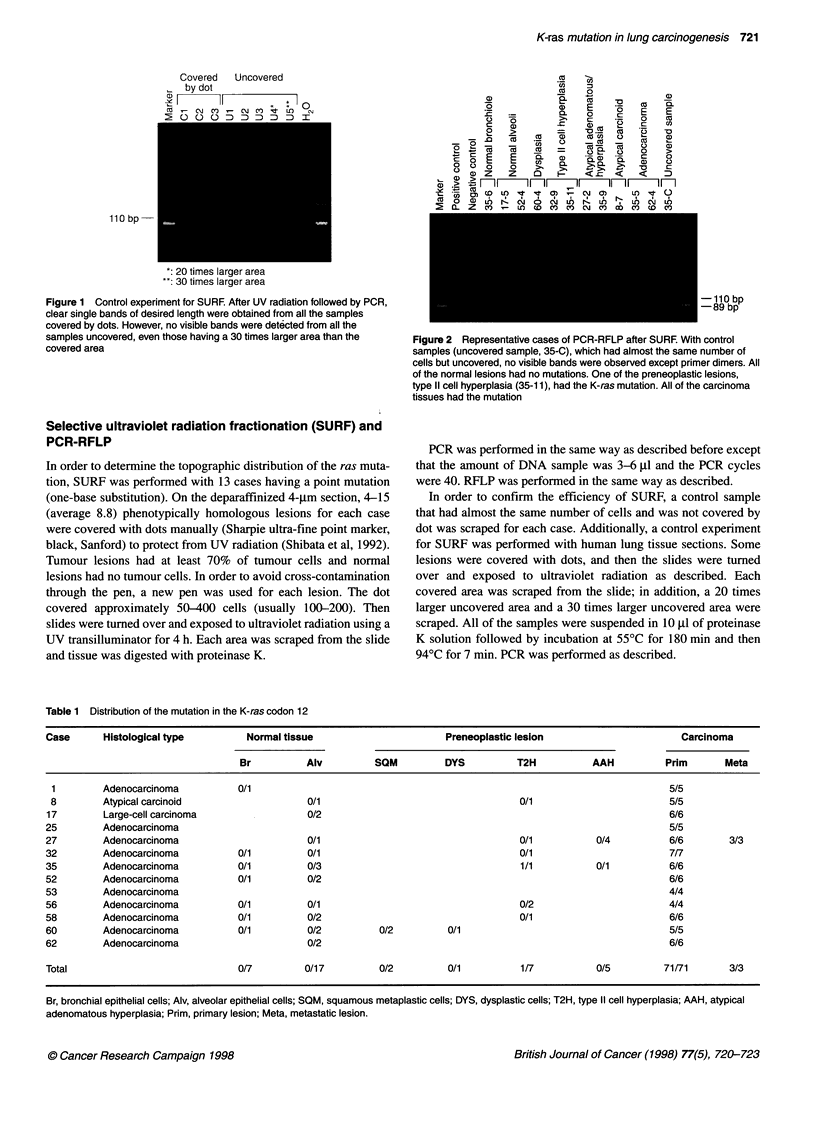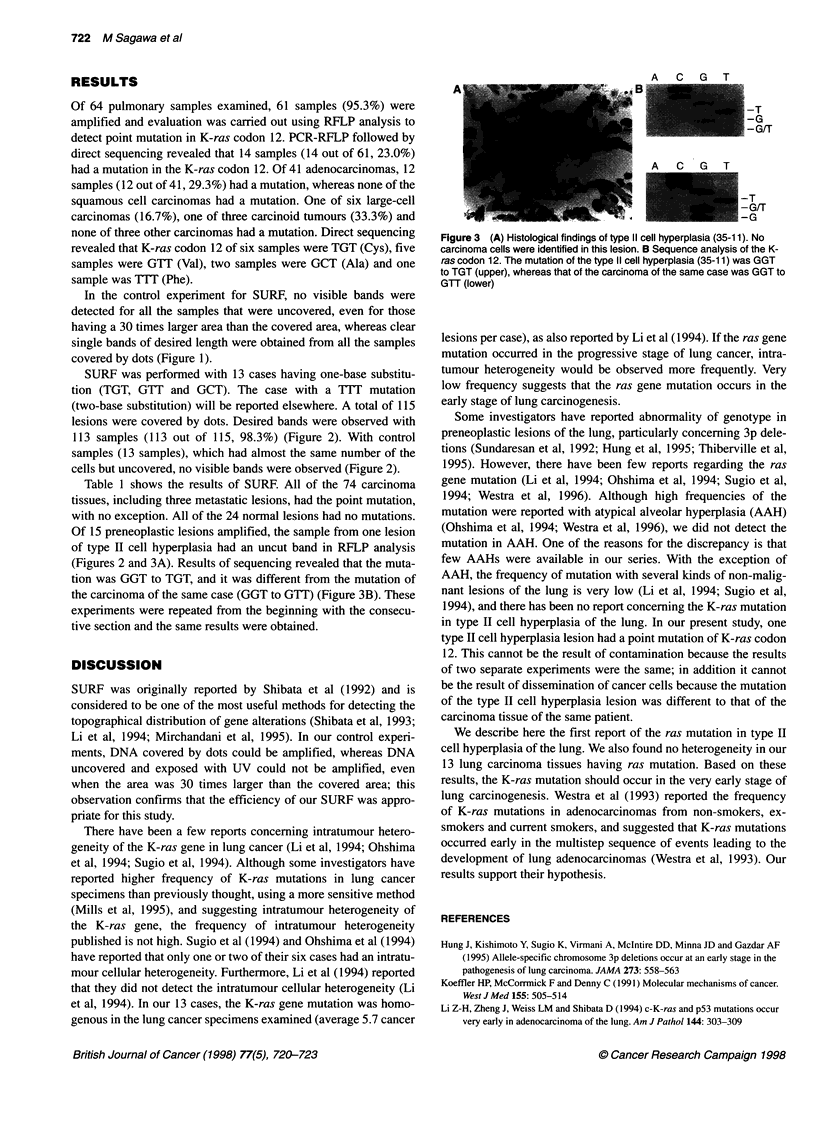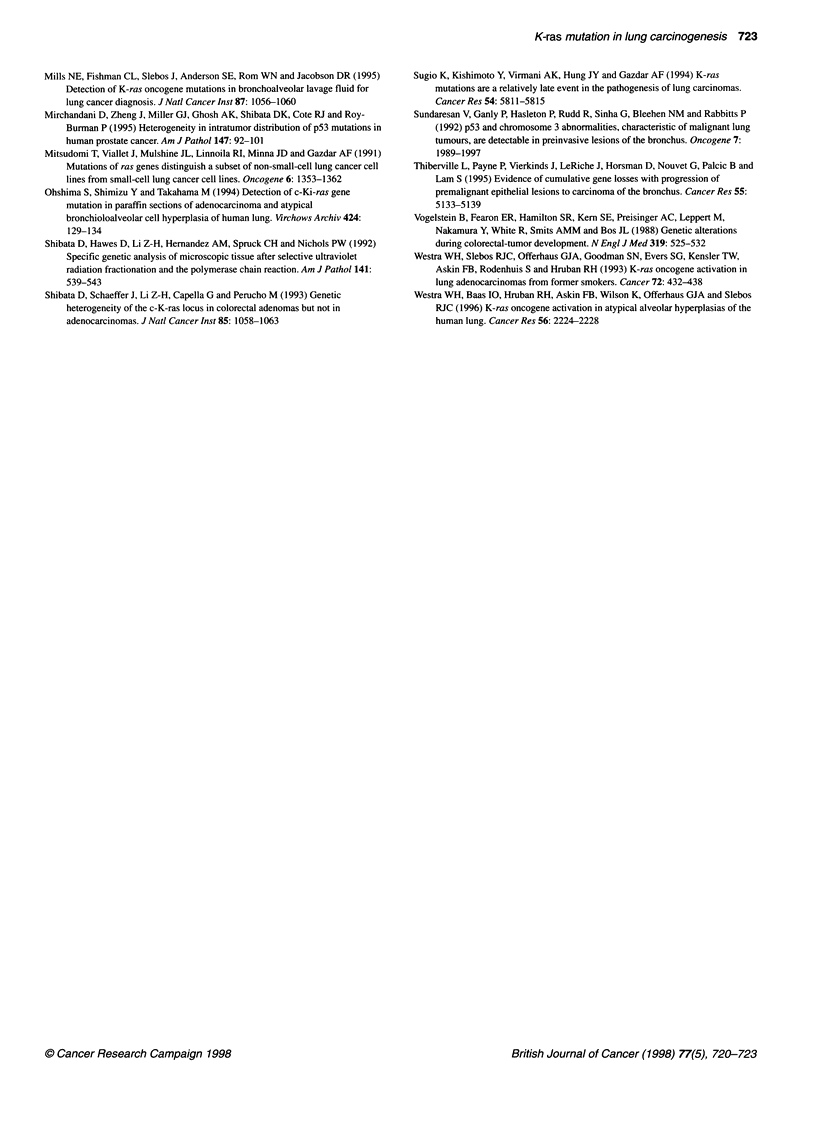# K-ras point mutation occurs in the early stage of carcinogenesis in lung cancer.

**DOI:** 10.1038/bjc.1998.118

**Published:** 1998-03

**Authors:** M. Sagawa, Y. Saito, S. Fujimura, R. I. Linnoila

**Affiliations:** Biomarkers and Prevention Research Branch, National Cancer Institute, Rockville, Maryland 20850, USA.

## Abstract

**Images:**


British Journal of Cancer (1998) 77(5), 720–723
© 1998 Cancer Research Campaign

### K-*ras* point mutation occurs in the early stage of carcinogenesis in lung cancer

**M Sagawa[1,2], Y Saito[2], S Fujimura[2] and RI Linnoila[1]**

[1]Biomarkers and Prevention Research Branch, National Cancer Institute, 9610 Medical Center Drive, Rockville, Maryland 20850, USA; [2]Department of Thoracic Surgery, Institute of Development, Aging and Cancer, Tohoku University, 4-1 Seiryo-machi, Aoba-ku, Sendai 980-77, Japan

**Summary** In order to determine the topographical distribution of the K-*ras* codon 12 mutations in carcinoma and preneoplastic lesions of the lung, selective ultraviolet radiation fractionation, as well as microdissection followed by polymerase chain reaction–restriction fragment length polymorphism (PCR-RELP), was performed. Fourteen of 61 samples amplified (23.0%) had a mutation in the K-*ras* codon 12. Of 41 adenocarcinoma, 12 samples (29.3%) had a mutation, whereas none of the squamous cell carcinomas had a mutation. One of six large-cell carcinomas, one of three carcinoid tumours and none of three other carcinomas had a mutation. Direct sequencing revealed that K-*ras* codon 12 of six samples were TGT (Cys), five samples were GTT (Val), two samples were GCT (Ala) and one sample was TTT (Phe). A total of 113 lesions of 13 cases covered by dot were amplified after UV radiation. All of 74 carcinoma lesions had the mutation, and intratumour heterogeneity was not observed. Of 39 non-malignant lesions, one type II cell hyperplasia had the mutation, which suggests that the K-*ras* mutation occurs in the early stage of carcinogenesis. The lack of intratumour heterogeneity supports the hypothesis.

**Keywords:** K-*ras*; lung cancer; preneoplastic lesion; genetic heterogeneity; selective ultraviolet radiation fractionation

Mutation in the K-*ras* gene is one of the most frequent gene alterations in human pancreatic cancers, colon cancers and adenocarcinomas of the lung (Vogelstein et al, 1988; Koeffler et al, 1991; Mitsudomi et al, 1991). Point mutation in the K-*ras* codon 12 is considered as an important genetic change in the carcinogenesis of adenocarcinoma of the lung. However, the topographical distribution of the K-*ras* point mutation in preneoplastic lesions of the lung is not fully understood, and the intratumour cellular heterogeneity in lung carcinoma tissues has not been reported satisfactorily. In the present study, we tried to determine the topographical distribution of the K-*ras* codon 12 mutations in carcinoma and preneoplastic lesions of the lung, using the selective ultraviolet radiation fractionation (SURF) method (Shibata et al, 1992) as well as microdissection followed by polymerase chain reaction–restriction fragment length polymorphism (PCR-RFLP).

### MATERIALS AND METHODS

### DNA preparation

Sixty-four lung carcinoma tissues resected at the NCI-Navy Hospital were fixed with 10% buffered formalin and embedded in paraffin. Two 4-µm sections were used for DNA extraction. After deparaffinization, only carcinoma tissue was scraped from the slide using a sterile pipette tip and 200 µl of proteinase K solution (400 µg $ml^{-1}$ proteinase K, 50 mM Tris, 1 mM EDTA, 0.5% Tween 20, pH 8.5) and was transferred to a tube followed by incubation at 55°C for 180 min and then 94°C for 7 min.

*Received 26 March 1997*
*Revised 14 July 1997*
*Accepted 21 July 1997*

*Correspondence to:* M Sagawa, Department of Thoracic Surgery, Institute of Development, Aging and Cancer, Tohoku University, 4-1 Seiryo-machi, Aoba-ku, Sendai 980-77, Japan

### PCR-RFLP and direct sequencing

Primers were designed to induce restriction site of *Ban* I (sense primer, 5′-CATGTTCTAATATAGTCACA-3′; antisense-RFLP primer, 5′-CAAGGCACTCTTGCCTACGGC-3′; synthesized by Midland Certified Reagent Company, Midland, TX, USA). PCR products can be cut by *Ban* I (99 base pairs and 21 base pairs) with wild-type DNA, whereas *Ban* I cannot cut the PCR products (110 base pairs) when the mutation in codon 12 exists.

Twenty-five microlitres of PCR lower mixture [300 µM of each dNTP, 6 mM of magnesium chloride, 2.5 µl of 10 × PCR buffer (500 mM potassium chloride, 200 mM Tris-HCl, pH 8.4, Gibco) and 0.5 µg of each primer] were placed into a 200-µl MicroAmp reaction tube (Perkin Elmer). To allow the 'hot start', Ampliwax (Perkin Elmer) was added and the tubes were heated to 80°C for 5 min to melt the wax. After the wax had hardened, 50 µl of PCR upper mixture [5 µl of 10 × PCR buffer, 2.5 units of *Taq* DNA polymerase (Gibco) and 10 µl of DNA sample] were added to the tubes. PCR was performed in a DNA thermal cycler (GeneAmp PCR System 9600, Perkin Elmer Cetus). PCR conditions were: 35–40 cycles of 94°C for 30 s, 52°C for 1 min, 68°C for 1 min, followed by 68°C for 10 min.

The condition of the digestion with *Ban* I was optimized in a pilot study, because the salt in the PCR mixture may have affected the efficiency of the enzyme. After thermal cycling, 20 µl of each reaction mixture was taken to another tube and 2 µl of *Ban* I (New England Biolabs) was added, followed by incubation at 37°C for 120 min and then 65°C for 5 min. Ten microlitres of each sample (with or without *Ban* I) were taken and electrophoresed through 4% Nu-Sieve 3:1 agarose gel.

Direct sequencing was performed with all the samples that had 110-bp bands after *Ban* I digestion and with some samples that did not have uncut bands for controls. Cycle sequencing kit (Gibco) was used for this purpose according to the manufacturer's instructions.

**Figure 1** Control experiment for SURF. After UV radiation followed by PCR, clear single bands of desired length were obtained from all the samples covered by dots. However, no visible bands were detected from all the samples uncovered, even those having a 30 times larger area than the covered area

**Figure 2** Representative cases of PCR-RFLP after SURF. With control samples (uncovered sample, 35-C), which had almost the same number of cells but uncovered, no visible bands were observed except primer dimers. All of the normal lesions had no mutations. One of the preneoplastic lesions, type II cell hyperplasia (35-11), had the K-*ras* mutation. All of the carcinoma tissues had the mutation

### Selective ultraviolet radiation fractionation (SURF) and PCR-RFLP

In order to determine the topographic distribution of the *ras* mutation, SURF was performed with 13 cases having a point mutation (one-base substitution). On the deparaffinized 4-μm section, 4–15 (average 8.8) phenotypically homologous lesions for each case were covered with dots manually (Sharpie ultra-fine point marker, black, Sanford) to protect from UV radiation (Shibata et al, 1992). Tumour lesions had at least 70% of tumour cells and normal lesions had no tumour cells. In order to avoid cross-contamination through the pen, a new pen was used for each lesion. The dot covered approximately 50–400 cells (usually 100–200). Then slides were turned over and exposed to ultraviolet radiation using a UV transilluminator for 4 h. Each area was scraped from the slide and tissue was digested with proteinase K.

PCR was performed in the same way as described before except that the amount of DNA sample was 3–6 μl and the PCR cycles were 40. RFLP was performed in the same way as described.

In order to confirm the efficiency of SURF, a control sample that had almost the same number of cells and was not covered by dot was scraped for each case. Additionally, a control experiment for SURF was performed with human lung tissue sections. Some lesions were covered with dots, and then the slides were turned over and exposed to ultraviolet radiation as described. Each covered area was scraped from the slide; in addition, a 20 times larger uncovered area and a 30 times larger uncovered area were scraped. All of the samples were suspended in 10 μl of proteinase K solution followed by incubation at 55°C for 180 min and then 94°C for 7 min. PCR was performed as described.

**Table 1** Distribution of the mutation in the K-*ras* codon 12

| Case | Histological type | Normal tissue | | Preneoplastic lesion | | | | Carcinoma | |
|---|---|---|---|---|---|---|---|---|---|
| | | Br | Alv | SQM | DYS | T2H | AAH | Prim | Meta |
| 1 | Adenocarcinoma | 0/1 | | | | | | 5/5 | |
| 8 | Atypical carcinoid | | 0/1 | | | 0/1 | | 5/5 | |
| 17 | Large-cell carcinoma | | 0/2 | | | | | 6/6 | |
| 25 | Adenocarcinoma | | | | | | | 5/5 | |
| 27 | Adenocarcinoma | | 0/1 | | | 0/1 | 0/4 | 6/6 | 3/3 |
| 32 | Adenocarcinoma | 0/1 | 0/1 | | | 0/1 | | 7/7 | |
| 35 | Adenocarcinoma | 0/1 | 0/3 | | | 1/1 | 0/1 | 6/6 | |
| 52 | Adenocarcinoma | 0/1 | 0/2 | | | | | 6/6 | |
| 53 | Adenocarcinoma | | | | | | | 4/4 | |
| 56 | Adenocarcinoma | 0/1 | 0/1 | | | 0/2 | | 4/4 | |
| 58 | Adenocarcinoma | 0/1 | 0/2 | | | 0/1 | | 6/6 | |
| 60 | Adenocarcinoma | 0/1 | 0/2 | 0/2 | 0/1 | | | 5/5 | |
| 62 | Adenocarcinoma | | 0/2 | | | | | 6/6 | |
| Total | | 0/7 | 0/17 | 0/2 | 0/1 | 1/7 | 0/5 | 71/71 | 3/3 |

Br, bronchial epithelial cells; Alv, alveolar epithelial cells; SQM, squamous metaplastic cells; DYS, dysplastic cells; T2H, type II cell hyperplasia; AAH, atypical adenomatous hyperplasia; Prim, primary lesion; Meta, metastatic lesion.

© Cancer Research Campaign 1998

### RESULTS

Of 64 pulmonary samples examined, 61 samples (95.3%) were amplified and evaluation was carried out using RFLP analysis to detect point mutation in K-*ras* codon 12. PCR-RFLP followed by direct sequencing revealed that 14 samples (14 out of 61, 23.0%) had a mutation in the K-*ras* codon 12. Of 41 adenocarcinomas, 12 samples (12 out of 41, 29.3%) had a mutation, whereas none of the squamous cell carcinomas had a mutation. One of six large-cell carcinomas (16.7%), one of three carcinoid tumours (33.3%) and none of three other carcinomas had a mutation. Direct sequencing revealed that K-*ras* codon 12 of six samples were TGT (Cys), five samples were GTT (Val), two samples were GCT (Ala) and one sample was TTT (Phe).

In the control experiment for SURF, no visible bands were detected for all the samples that were uncovered, even for those having a 30 times larger area than the covered area, whereas clear single bands of desired length were obtained from all the samples covered by dots (Figure 1).

SURF was performed with 13 cases having one-base substitution (TGT, GTT and GCT). The case with a TTT mutation (two-base substitution) will be reported elsewhere. A total of 115 lesions were covered by dots. Desired bands were observed with 113 samples (113 out of 115, 98.3%) (Figure 2). With control samples (13 samples), which had almost the same number of the cells but uncovered, no visible bands were observed (Figure 2).

Table 1 shows the results of SURF. All of the 74 carcinoma tissues, including three metastatic lesions, had the point mutation, with no exception. All of the 24 normal lesions had no mutations. Of 15 preneoplastic lesions amplified, the sample from one lesion of type II cell hyperplasia had an uncut band in RFLP analysis (Figures 2 and 3A). Results of sequencing revealed that the mutation was GGT to TGT, and it was different from the mutation of the carcinoma of the same case (GGT to GTT) (Figure 3B). These experiments were repeated from the beginning with the consecutive section and the same results were obtained.

**Figure 3** (**A**) Histological findings of type II cell hyperplasia (35-11). No carcinoma cells were identified in this lesion. **B** Sequence analysis of the K-*ras* codon 12. The mutation of the type II cell hyperplasia (35-11) was GGT to TGT (upper), whereas that of the carcinoma of the same case was GGT to GTT (lower)

### DISCUSSION

SURF was originally reported by Shibata et al (1992) and is considered to be one of the most useful methods for detecting the topographical distribution of gene alterations (Shibata et al, 1993; Li et al, 1994; Mirchandani et al, 1995). In our control experiments, DNA covered by dots could be amplified, whereas DNA uncovered and exposed with UV could not be amplified, even when the area was 30 times larger than the covered area; this observation confirms that the efficiency of our SURF was appropriate for this study.

There have been a few reports concerning intratumour heterogeneity of the K-*ras* gene in lung cancer (Li et al, 1994; Ohshima et al, 1994; Sugio et al, 1994). Although some investigators have reported higher frequency of K-*ras* mutations in lung cancer specimens than previously thought, using a more sensitive method (Mills et al, 1995), and suggesting intratumour heterogeneity of the K-*ras* gene, the frequency of intratumour heterogeneity published is not high. Sugio et al (1994) and Ohshima et al (1994) have reported that only one or two of their six cases had an intratumour cellular heterogeneity. Furthermore, Li et al (1994) reported that they did not detect the intratumour cellular heterogeneity (Li et al, 1994). In our 13 cases, the K-*ras* gene mutation was homogenous in the lung cancer specimens examined (average 5.7 cancer lesions per case), as also reported by Li et al (1994). If the *ras* gene mutation occurred in the progressive stage of lung cancer, intratumour heterogeneity would be observed more frequently. Very low frequency suggests that the *ras* gene mutation occurs in the early stage of lung carcinogenesis.

Some investigators have reported abnormality of genotype in preneoplastic lesions of the lung, particularly concerning 3p deletions (Sundaresan et al, 1992; Hung et al, 1995; Thiberville et al, 1995). However, there have been few reports regarding the *ras* gene mutation (Li et al, 1994; Ohshima et al, 1994; Sugio et al, 1994; Westra et al, 1996). Although high frequencies of the mutation were reported with atypical alveolar hyperplasia (AAH) (Ohshima et al, 1994; Westra et al, 1996), we did not detect the mutation in AAH. One of the reasons for the discrepancy is that few AAHs were available in our series. With the exception of AAH, the frequency of mutation with several kinds of non-malignant lesions of the lung is very low (Li et al, 1994; Sugio et al, 1994), and there has been no report concerning the K-*ras* mutation in type II cell hyperplasia of the lung. In our present study, one type II cell hyperplasia lesion had a point mutation of K-*ras* codon 12. This cannot be the result of contamination because the results of two separate experiments were the same; in addition it cannot be the result of dissemination of cancer cells because the mutation of the type II cell hyperplasia lesion was different to that of the carcinoma tissue of the same patient.

We describe here the first report of the *ras* mutation in type II cell hyperplasia of the lung. We also found no heterogeneity in our 13 lung carcinoma tissues having *ras* mutation. Based on these results, the K-*ras* mutation should occur in the very early stage of lung carcinogenesis. Westra et al (1993) reported the frequency of K-*ras* mutations in adenocarcinomas from non-smokers, ex-smokers and current smokers, and suggested that K-*ras* mutations occurred early in the multistep sequence of events leading to the development of lung adenocarcinomas (Westra et al, 1993). Our results support their hypothesis.

### REFERENCES

Hung J, Kishimoto Y, Sugio K, Virmani A, McIntire DD, Minna JD and Gazdar AF (1995) Allele-specific chromosome 3p deletions occur at an early stage in the pathogenesis of lung carcinoma. *JAMA* **273**: 558–563

Koeffler HP, McCormick F and Denny C (1991) Molecular mechanisms of cancer. *West J Med* **155**: 505–514

Li Z-H, Zheng J, Weiss LM and Shibata D (1994) c-K-*ras* and p53 mutations occur very early in adenocarcinoma of the lung. *Am J Pathol* **144**: 303–309

© Cancer Research Campaign 1998

Mills NE, Fishman CL, Slebos J, Anderson SE, Rom WN and Jacobson DR (1995) Detection of K-*ras* oncogene mutations in bronchoalveolar lavage fluid for lung cancer diagnosis. *J Natl Cancer Inst* **87**: 1056–1060

Mirchandani D, Zheng J, Miller GJ, Ghosh AK, Shibata DK, Cote RJ and Roy-Burman P (1995) Heterogeneity in intratumor distribution of p53 mutations in human prostate cancer. *Am J Pathol* **147**: 92–101

Mitsudomi T, Viallet J, Mulshine JL, Linnoila RI, Minna JD and Gazdar AF (1991) Mutations of *ras* genes distinguish a subset of non-small-cell lung cancer cell lines from small-cell lung cancer cell lines. *Oncogene* **6**: 1353–1362

Ohshima S, Shimizu Y and Takahama M (1994) Detection of c-Ki-*ras* gene mutation in paraffin sections of adenocarcinoma and atypical bronchioloalveolar cell hyperplasia of human lung. *Virchows Archiv* **424**: 129–134

Shibata D, Hawes D, Li Z-H, Hernandez AM, Spruck CH and Nichols PW (1992) Specific genetic analysis of microscopic tissue after selective ultraviolet radiation fractionation and the polymerase chain reaction. *Am J Pathol* **141**: 539–543

Shibata D, Schaeffer J, Li Z-H, Capella G and Perucho M (1993) Genetic heterogeneity of the c-K-ras locus in colorectal adenomas but not in adenocarcinomas. *J Natl Cancer Inst* **85**: 1058–1063

Sugio K, Kishimoto Y, Virmani AK, Hung JY and Gazdar AF (1994) K-*ras* mutations are a relatively late event in the pathogenesis of lung carcinomas. *Cancer Res* **54**: 5811–5815

Sundaresan V, Ganly P, Hasleton P, Rudd R, Sinha G, Bleehen NM and Rabbitts P (1992) p53 and chromosome 3 abnormalities, characteristic of malignant lung tumours, are detectable in preinvasive lesions of the bronchus. *Oncogene* **7**: 1989–1997

Thiberville L, Payne P, Vierkinds J, LeRiche J, Horsman D, Nouvet G, Palcic B and Lam S (1995) Evidence of cumulative gene losses with progression of premalignant epithelial lesions to carcinoma of the bronchus. *Cancer Res* **55**: 5133–5139

Vogelstein B, Fearon ER, Hamilton SR, Kern SE, Preisinger AC, Leppert M, Nakamura Y, White R, Smits AMM and Bos JL (1988) Genetic alterations during colorectal-tumor development. *N Engl J Med* **319**: 525–532

Westra WH, Slebos RJC, Offerhaus GJA, Goodman SN, Evers SG, Kensler TW, Askin FB, Rodenhuis S and Hruban RH (1993) K-*ras* oncogene activation in lung adenocarcinomas from former smokers. *Cancer* **72**: 432–438

Westra WH, Baas IO, Hruban RH, Askin FB, Wilson K, Offerhaus GJA and Slebos RJC (1996) K-*ras* oncogene activation in atypical alveolar hyperplasias of the human lung. *Cancer Res* **56**: 2224–2228

© Cancer Research Campaign 1998